# Influence of vitamin D supplementation on plasma lipid profiles: A meta-analysis of randomized controlled trials

**DOI:** 10.1186/1476-511X-11-42

**Published:** 2012-03-20

**Authors:** Hao Wang, Ning Xia, Yang Yang, Dao-Quan Peng

**Affiliations:** 1 Department of Cardiology, the Second Xiangya Hospital, Central South University, Changsha 410011, Hunan, People's Republic of China; 2 Department of Pulmonary, Xiangya Hospital, Central South University, Changsha 410011, Hunan, People's Republic of China

**Keywords:** Vitamin D, lipids, cardiovascular disease, meta-analysis

## Abstract

Observational studies have shown that low serum levels of vitamin D have been associated with an atherogenic lipid profile. However, the intervention studies gave divergent results. We conducted a meta-analysis of randomized controlled trials that evaluated the effects of vitamin D supplementation on blood lipids. A systematic literature search was conducted via MEDLINE, Cochrane library, and EMBASE for randomized controlled clinical trials assessing the effects of vitamin D supplementation on lipids. The mean change in total cholesterol (TC), low-density lipoprotein cholesterol (LDL-C), high-density lipoprotein cholesterol (HDL-C) and triglycerides (TG) from baseline was treated as a continuous variable. In all, 12 clinical trials consisting of 1346 participants were included in the analysis. The pooled estimate of effect for vitamin D supplementation on LDL-C was 3.23 mg/dl (95% confidence interval, 0.55 to 5.90 mg/dl). No statistically significant effects for vitamin D supplementation were observed for TC, HDL-C and TG (differences in means were 1.52 mg/dl (-1.42 to 4.46 mg/dl), -0.14 mg/dl (-0.99 to 0.71 mg/dl) and -1.92 mg/dl (-7.72 to 3.88 mg/dl) respectively). The lipid modulating effects of vitamin D supplementation should be further investigated though large-scale, randomized trials with adequate doses which can effectively elevated the active form of vitamin D in plasma and with proper population which has hyperlipemia as an inclusion criterion.

## Introduction

Cardiovascular disease (CVD) remains the leading cause of death and disability in the world [1]. Although cardiovascular mortality rates have declined in some high-income countries, more than 17 million people died from CVD in 2008 and it is estimated that by 2030, almost 23.6 million people will die from CVD. Thus, extraordinary effort has been devoted to determining the modifying risk factors to prevent atherosclerosis, the main cause of CVD. There is now increasing evidence that vitamin D, beyond its well-known effects on bone metabolism, also plays an important role in the development of CVD [2]. Epidemiologic studies have shown that vitamin D deficiency was closely associated with increased risk of major adverse CVD events [3,4]. Furthermore, randomized intervention trials showed a tendency towards a reduction in CVD risk with vitamin D supplementation, though the tendency is statistically nonsignificant [5]. Taking into account that vitamin D deficiency is highly prevalent across the world while vitamin D supplementation is simple, safe, and inexpensive [6], the deficiency of vitamin D may be a common and easily treatable risk factor for CVD prevention [7].

There are several possible mechanisms contributing to the association between vitamin D and CVD, such as insulin sensitivity, parathyroid hormone elevation and inflammation [8]. It is reasonable that dyslipidemia should also be considered as a potential link because dyslipidemia is a well-described independent risk factor for CVD. Observational studies have indicated that high 25-hydroxyvitamin D [25(OH)D] levels were associated with a favorable serum lipid profile [9]. However, a solid rationale for such association is difficult to determine unless there is an effect of vitamin D supplementation on serum lipids in placebo-controlled randomized trials. Unfortunately, the intervention studies gave divergent results that some showing a positive and some a negative effect [9]. Therefore, we conducted a meta-analysis of randomized controlled trials to evaluate the potential effect of vitamin D supplementation on serum lipid profiles.

## Methods

### Search strategy and study selection

A systematic search of the literature published prior to November 2011 was conducted in MEDLINE, Cochrane library, and EMBASE to identify all articles related to randomized controlled trials examining the effect of vitamin D supplementation on the blood lipid profile. The following key words were used in all fields: (vitamin D OR 25-hydroxyvitamin D OR vitamin D3 OR cholecalciferol OR ergocalciferol OR calcifediol OR calcitriol) AND (lipids OR cholesterol OR triglycerides OR HDL OR LDL OR apolipoprotein A OR apolipoprotein B). No language and time restrictions were imposed and no attempt was made to include abstracts or unpublished studies. Then, duplicate citations were removed. In addition, a manual search of references from primary or review articles was performed to identify relevant trials.

Trials were included in the analysis if they were randomized controlled trials of vitamin D in human participants in which the mean changes of total cholesterol (TC), low-density lipoprotein cholesterol (LDL-C), high-density lipoprotein cholesterol (HDL-C) and triglycerides (TG) concentration, along with standard deviation, were reported for the intervention and control groups. Studies where vitamin D was combined with calcium were only included if the placebo group was given the same calcium supplement. Furthermore, we excluded studies in which vitamin intake was mixed with other dietary treatments or drugs. We excluded studies that focused on the patients with non-cardiovascular diseases, such as chronic kidney disease, hemodialysis states and rheumatoid arthritis for the reason that these diseases have remarkable impact on lipids profile and might confused the effects of vitamin D supplement on lipids. In trials for which there was more than one published report on the same population of patients, the most recent publication was selected for analysis. Trials involving more than one intervention group were included by entering each pair-wise comparison into the meta-analysis as separate trials, but with the repeated control groups' sample size divided out evenly among the comparisons.

### Data extraction and quality assessment

Two investigators (HW and NX) performed the data extraction independently. In case of disagreement, a third investigator was consulted (DQP). Discrepancies were resolved by consensus. The following information was abstracted from eligible articles: first author's name; year of publication; number and age range of participants; study design; vitamin D type and dose used; duration of study; health conditions of the study population. We also extracted information on the baseline and final concentrations (or net changes) of serum total cholesterol (TC), LDL cholesterol, HDL cholesterol, and triglycerides (TG).

The quantitative 5-point Jadad score system was used to assess the strength of included studies [10]. This included independent assessments by two investigators (HW and NX) of factors referring the description of randomization, double blinding, and drop-outs.

### Data synthesis and statistical analysis

The mean change in each lipid parameter was the outcome of interest in this meta-analysis. Studies that reported results in mmol/l were converted to mg/dl. The conversion factor was 1 mg/dl = 0.0259 mmol/l for TC, HDL and LDL; and 1 mg/dl = 0.0113 mmol/l for TG. Standard errors were converted to standard deviation for the analyses. Studies not reporting standard deviations or standard errors were excluded.

The mean change in each lipid parameter from baseline was treated as a continuous variable. Some studies reported only mean values with standard deviations of the baseline and endpoint for the paired groups. In this condition, net changes for the lipid parameters were calculated as the difference (intervention minus control) of the changes (endpoint minus baseline) in mean values. Standard deviation was calculated from the variances of baseline and endpoint using a correlation coefficient of 0.5[11].

$$\begin{array}{l} SD(difference)=[(SD^{2}(intervention)+SD^{2}(control)\\ {-2\times 0.5\times SD(intervention)\times SD(control)]}^{1/2} \end{array}$$

Statistical heterogeneity was addressed using Cochrane Q test, and the magnitude of heterogeneity was estimated by *I^2 ^*statistic. Visual inspection of funnel plots was used to assess for the presence of publication bias.

## Results

**Figure 1:**
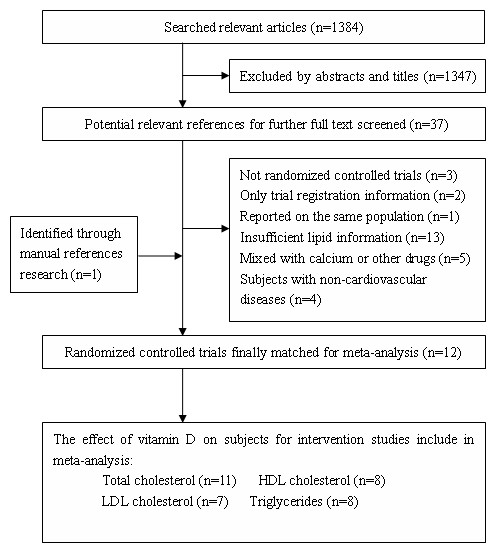
**Flow diagram of study selection process**.

A total of 1384 articles were found in our initial search, 1347 of which could be excluded by screening the titles or abstracts. A further 13 articles were excluded because they did not report sufficient details on blood lipid parameters. Among them, one study met all our inclusion criteria but could not be incorporated into the meta-analysis because it reported quartiles instead of variance [12]. 5 more trials were excluded because vitamin D was a part of mixed intervention [13-17]. Other 4 studies were not included because they focused on the hemodialysis patients [18-21]. A flow diagram on articles selection for this meta-analysis is shown in Figure 1.

In all, 10 articles matched for our inclusion criterion [22-31]. The studies performed by Jorde et al. [27] and Witham et al. [28] involved two intervention groups respectively and we included each pair-wise comparison into the meta-analysis as separate trials. Therefore, 12 randomized controlled trials were included in our meta-analysis. Characteristics of included studies are presented in Table 1. All of the trials were double-blind, randomized and controlled clinical studies with parallel design. Eight of the studies obtained a Jadad score of > 3. The selected trials involved 1346 individuals in total, ranging in age from 18 to 80 years. Four of the studies [23-25,31] were conducted on healthy subjects and three others [26,27,30] were carried out on obese subjects. Other two studies [22,28] involved diabetic patients and one study [29] involved patients with a history of stroke. Adherence varied between 75% and 100%, with eleven out of twelve trials reporting adherence of 80-100%.

**Table 1:** Characteristics of the enrolled references in the meta-analysis

Trial	Design	Sample size	Age range (yrs)	Population characteristics	Duration	Intervention	Control	Outcomes	Jadad quality grades (0-5)
Ljunghall et al. (1987)	Parallel	65	61-65	Middle-aged men with impaired glucose tolerance; Swedish	12 weeks	0.75 ug alpha-calcidol, daily	placebo	TC, HDL, TG	1
Heikkinen et al. (1997)	Parallel	178	47-56	Postmenopausal women; Finnish	3 years	300 IU cholecalciferol+500 mg calcium lactate, daily	500 mg calcium lactate	TC, LDL, HDL, TG	2
Pfeifer et al. (2001)	Parellel	148	74 ± 1	Elderly women with low vitamin D status; German	8 weeks	800 IU vitamin D3+1200 mg calcium, daily	1200 mg calcium	TC	4
Nagpal et al. (2009)	Parallel	71	≥ 35	Middle-aged healthy men with central obesity; Indian	42 days	120000 IU cholecalciferol, fortnightly	placebo	TC, LDL, HDL, TG	5
Zittermann et al. (2009)	Parallel	165	48 ± 10	Healthy overweight subjects; German	1 year	3332 IU cholecalciferol, daily	placebo	LDL, HDL, TG	5
Jorde et al. (a/b) (2010)	Parallel	216/226	21-70	Overweight and obese subjects; Norwegian	1 year	20000/40000 IU cholecalciferol weekly +500 mg calcium daily	500 mg calcium	TC, LDL, HDL, TG	4
Witham et al. (a/b) (2010)	Parallel	40/39	65 ± 10	Patients with type 2 diabetes; Scottish	16 weeks	100000/200000 IU vitamin D3, once	placebo	TC	5
Witham et al. (2010)	Parallel	58	67 ± 10	Patients with a history of stroke; Scottish	16 weeks	100000 IU ergocalciferol once	placebo	TC	5
Maki et al. (2011)	Parallel	60	18-79	Subjects with high waist circumference; American	8 weeks	1200 IU cholecalciferol+MVM (multivitamin and mineral), daily	MVM	TC, LDL, HDL, TG	5
Sai et al. (2011)	Parallel	213	65-77	Postmenopausal women; American	3 years	0.5 ug calcitriol, daily	placebo	TC, LDL, HDL, TG	4

TC, total cholesterol; HDL, high density lipoprotein cholesterol; LDL, low density lipoprotein cholesterol; TG, triglycerides

Vitamin D3/cholecalciferol was used in seven studies and other studies used alpha-calcidol, calcitriol or ergocalciferol. In studies which administered a daily dose of vitamin D supplement, the dose ranged from 300 IU to 3332 IU. Furthermore, two trials administered a single dose of vitamin D supplement and another trial administered a supplement fortnightly [25,28,29]. The duration of the intervention ranged from 42 days to 3 years. Calcium supplementation was used in both treatment and placebo groups in four trials, and the dose varied between 500 mg/day and 1200 mg/day. Data of initial 25(OH)D levels as well as achieved 25(OH)D levels were available in ten of the trials. Most of the studies showed that the intake of vitamin D supplements resulted in increases in serum 25(OH)D levels (Table 2).

**Table 2:** Serum levels of 25-hydroxyvitamin D in randomized trials with Vitamin D supplements

Trial	Mean serum 25-hydroxyvitamin D3 (nmol/L)			
	
	Intervention group		Control group	
	
	Initial levels	Achieved levels	Initial levels	Achieved levels
Ljunghall et al. (1987)	75.5	85.7	79.6	110.2
Pfeifer et al. (2001)	25.65	64.84	24.63	44.36
Nagpal et al. (2009)	36.5	71.6	30.0	30.6
Zittermann et al. (2009)	30.0	85.5	30.3	42.0
Jorde et al. (a). (2010)	56.7	99.5	58.8	57.2
Jorde et al. (b). (2010)	58.7	138.0	58.8	57.2
Witham et al. (a). (2010)	41.0	63.0	45.0	54.0
Witham et al. (b). (2010)	48.0	79.0	45.0	54.0
Witham et al. (2010)	38.7	51.0	37.8	40.0
Maki et al. (2011)	64.4	76.1	67.9	66.7

**Figure 2:**
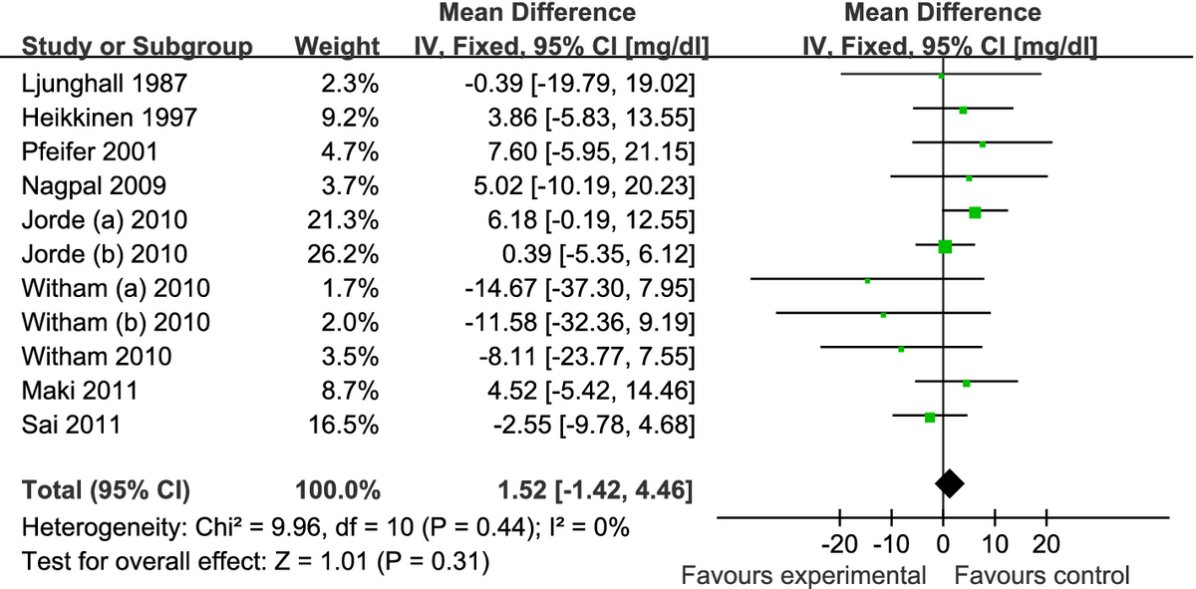
**Forest plots depicting the effect of vitamin D supplement on low-density lipoprotein (LDL) cholesterol**. IV, inverse variance; fixed, fixed effects model; CI, confidence interval.

**Figure 3:**
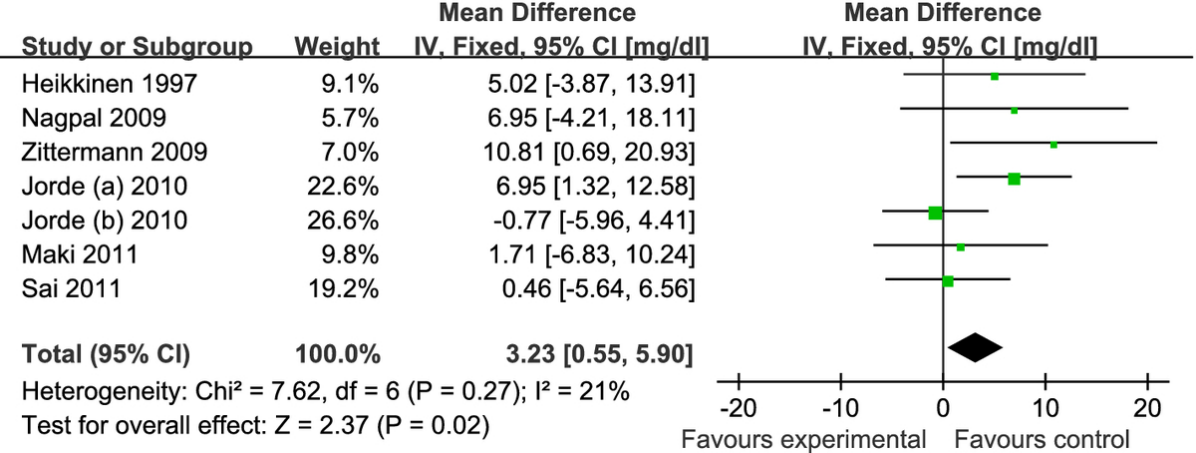
**Forest plots depicting the effect of vitamin D supplement on total cholesterol**. IV, inverse variance; fixed, fixed effects model; CI, confidence interval.

**Figure 4:**
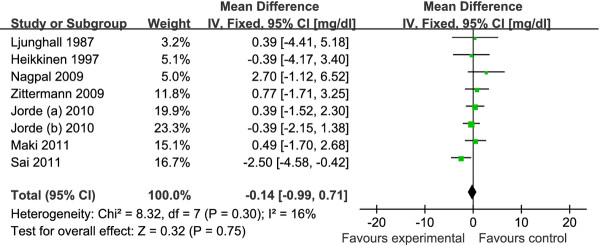
**Forest plots depicting the effect of vitamin D supplement on high-density lipoprotein (HDL) cholesterol**. IV, inverse variance; fixed, fixed effects model; CI, confidence interval.

**Figure 5:**
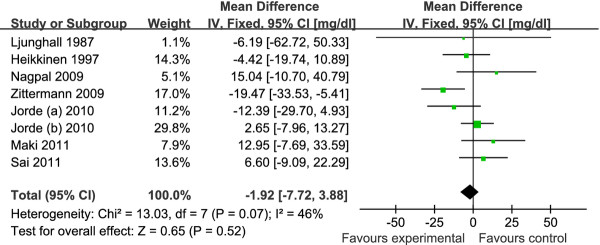
**Forest plots depicting the effect of vitamin D supplement on triglycerides**. IV, inverse variance; fixed, fixed effects model; CI, confidence interval.

The pooled mean net change in LDL-C concentration (95% CI) comparing vitamin D supplement with placebo was statistically significant as 3.23 mg/dl (0.55, 5.90) (Figure 2). Corresponding changes for TC, HDL-C and TG were 1.52 mg/dl (-1.42, 4.46) (Figure 3), -0.14 mg/dl (-0.99, 0.71) (Figure 4) and -1.92 mg/dl (-7.72, 3.88) (Figure 5), respectively. No statistical heterogeneity was observed in any of the lipid parameter analyses. Total cholesterol, LDL-C, HDL-C and triglycerides had *I^2 ^*values of 0%, 21%, 16% and 46%, respectively.

Subgroup analysis were carried out with the data segregated by location (high latitude vs. middle and low latitude areas), interventional duration (> 1 year and ≤ 1 year), and obese vs. non-obese participants (define according to the body mass index) (Table 3). In three studies [26,27,30] carried out on obese subjects whose body mass index was greater than 30 kg/m^2^, pooled mean net change of LDL-C was significant increase (3.46 mg/dl, 0.17 to 6.76); while none of the results was statistically significant in normal weight subjects. Studies with longer duration (> 1 year) [23,31] showed a significant reduction in HDL-C (-2.01 mg/dl, -3.83 to -0.18) while trials of short duration (≤ 1 year) showed a significant increase in LDL-C (3.74 mg/dl, 0.58 to 6.90). Analysis by location in which studies were carried out revealed nonsignificant results. Sensitivity analysis that excluded the lower quality studies [22,23] showed similar effects of vitamin D supplement on lipid profiles (Table 3).

**Table 3:** Results of subgroup and sensitivity analyses for evaluating the effect of vitamin D on lipid profile

Variables	Total cholesterol	LDL-C	HDL-C	Triglycerides
	
	Mean difference (mg/dl) with 95% confidence interval and P value			
Weight of subjects				
Obesity[26,27,30]	3.22 [-0.70, 7.14] P = 0.11	3.46 [0.17, 6.76] **P = 0.04**	0.22 [-0.80, 1.23] P = 0.68	-4.38 [-11.52, 2.76] P = 0.23
Normal[22-25,28,29]	-0.68 [-5.12, 3.77] P = 0.77	2.77 [-1.82, 7.36] P = 0.24	-0.97 [-2.53, 0.59] P = 0.22	2.83 [-7.10, 12.76] P = 0.58
Interventional duration				
> 1 year[23,31]	-0.26 [-6.05, 5.54] P = 0.93	1.92 [-3.11, 6.95] P = 0.45	-2.01 [-3.83, -0.18] **P = 0.03**	0.96 [-10.01, 11.92] P = 0.86
≤ 1 year[22,24-30]	2.13 [-1.28, 5.54] P = 0.22	3.74 [0.58, 6.90] **P = 0.02**	0.38 [-0.58, 1.34] P = 0.44	-3.04 [-9.87, 3.79] P = 0.38
Location of studies				
High latitude area [22,23,27]	2.99 [-0.84, 6.81] P = 0.13	3.12 [-0.39, 6.63] P = 0.08	-0.04 [-1.23, 1.15] P = 0.95	-2.30 [-10.02, 5.42] P = 0.56
Middle and low latitude area[24-26,28-31]	-0.60 [-5.20, 3.99] P = 0.8	3.38 [-0.76, 7.52] P = 0.11	-0.24 [-1.47, 0.98] P = 0.7	-1.43 [-10.21, 7.34] P = 0.75
Sensitivity analyses				
Excluding low quality studies (Jadad < 4) [22,23]	1.32 [-1.80,4.45] P = 0.41	3.05 [0.24, 5.86] **P = 0.03**	-0.14 [-1.03, 0.75] P = 0.75	-1.44 [-7.74, 4.86] P = 0.65

The potential publication bias was examined by plotting sample size versus mean net change for lipid profiles of the studies included in our analysis. For all lipid profiles, the plots showed a typical funnel shape and no publication bias were found.

## Discussion

In our meta-analysis of randomized controlled trials, vitamin D supplementation provided a statistically significant increase in LDL-C (3.23 mg/dl). There was also a tendency towards an increase in TC (1.52 mg/dl) with supplementation of vitamin D, and the reductions in HDL-C (-0.14 mg/dl) and TG (-1.92 mg/dl) were both nonsignificant. The effect of vitamin D supplement on serum LDL-C levels seemed more significant in obese subjects and in studies with relatively shorter durations, while studies with longer durations only showed a significant reduction in HDL-C levels (-2.01 mg/dl). To our knowledge, this is the first meta-analysis looking at the effect of vitamin D supplement on lipid profiles. Two previous systematic reviews summarized the effect of vitamin D on serum lipid profiles [9,32], however, no attempt to meta-analyze the data was made. By pooling information from all qualified randomized controlled studies, the results provided here are more precise and powerful than those from the individual studies.

Observational studies have shown that high serum 25(OH)D concentrations are associated with a favorable lipid profile [9]. In the study by Jorde et al. [33] who included 8018 nonsmoking subjects in the cross-sectional study, there were highly significant positive associations between serum 25(OH)D and serum TC, HDL-C and LDL-C, and significant negative associations between serum 25(OH)D and both LDL-C/HDL-C ratio and TG after adjustment for gender, age, BMI and month of blood sampling. In consistent with this, an increase in serum 25(OH)D was associated with a significant decrease of serum TG in the longitudinal study involving 1762 nonsmoking subjects. Although these findings are provocative, it is important to understand the inherent limitations of observational studies. Associations found in cross-sectional studies are no proof of a causal relationship. Some common factors may be attributed to both the high serum 25(OH)D levels and favorable lipid profile. Individuals with habits of exercising outside frequently and eating nutritious food, which would elevate 25(OH)D levels, may have other healthy habits which could favorably affect lipid profiles. Therefore, intervention studies are needed to detect a causal relationship between 25(OH)D levels and lipids.

So far merely a few such intervention studies have been reported and the results provided by them are divergent. In addition, these studies are heterogeneous with respect to vitamin D dose, study duration, and the characteristics of subjects. In particular, the wide variation in the amount and formulation of supplemental vitamin D may be the most important contributor to the heterogeneity found in our results. To achieve 25(OH)D levels above 75 nmol/L, the recommended level for several health outcomes [34], the daily intake of at least 1000 IU (40 ug) vitamin D (cholecalciferol) would be required. Most of the studies included in this analysis used a high dosage of vitamin D (>/= 1000 IU) resulting in significantly elevated 25(OH)D levels after the treatment (Table 2). Notably, as the active form of vitamin D, 1,25-dihydroxyvitamin D (1,25-D) is considered to be more appropriate than 25(OH)D for assessing the links between vitamin D and lipids [35]. It has been shown that 25(OH)D and 1,25-D had similar but independent biological effects. Unfortunately, few studies reported baseline 1,25-D levels and its changes after the intervention. As a result, it is not possible to evaluate the treatment effects of vitamin D on 1,25-D and the relationship between 1,25-D and lipids. Furthermore, not all included studies used vitamin D3 as supplementation. One study [29] used vitamin D2 and two other studies [22,31] used alpha-calcidol. Vitamin D2 is less bioactive than vitamin D3, whereas alpha-calcidol is a direct precursor of 1,25-D. These differences make a direct comparison of study results difficult.

Subgroup analyses by duration of intervention revealed that vitamin D treatment has a more obvious effect on LDL-C in the shorter duration studies. This may be because longer duration studies are associated with poor compliance of the subjects. On the other hand, studies with longer durations showed a significant reduction in HDL-C levels. It is in agreement with the fact that in vitamin D receptor knockout mice, there are higher HDL-C levels and hepatic apoA-I mRNA expression relative to wild type mice [36]. Experiments in cultured human hepatocytes also showed the metabolites of vitamin D had a potent inhibitory effect on apoA-I production and decreased both apoA-I secretion and apoA-I mRNA levels [37,38].

It is reasonable to speculate that the treatment effects of vitamin D are influenced by its baseline levels and the increment in blood levels. However, it was not possible to assess the effect of baseline vitamin D status on lipids profile from this meta-analysis, as the populations among individual studies were heterogeneous. In addition, different geographical latitudes of the study sites might have further complicated the issue of baseline value of vitamin D, but the subgroup analysis by study sites revealed nonsignificant results.

Although we have excluded the studies that focused on patients in hemodialysis states, other confounders that may have an unexpected influence on lipids could not be eliminated. Among them, obesity is always associated with dyslipidemia which includes high levels of TG and LDL-C and low levels of HDL-C [39]. Additionally, obesity may have an effect on vitamin D metabolism because adipose tissue in obese subjects preferentially uptakes vitamin D [40]. Therefore, we conducted a subgroup analyses by the weight of subjects and it showed a greater increase in serum LDL-C concentrations in obese subjects. However, there was no significant effect in the normal weight subjects.

Our review has several limitations. First, we found few eligible studies and none of them were sufficiently powered because they had relatively small numbers of participants. Second, most participants included in our studies were non-Hispanic White and elderly which limits the applicability of our results to other groups in the whole population. Third, as with any meta-analysis, the potential for publication bias needs to be discussed. However, the visual inspection of funnel plots suggests that the presence of publication bias in this meta-analysis is less likely. Finally, none of the studies included in our analysis were specifically designed to evaluate the effect of vitamin D on serum lipids and none had hyperlipemia as an inclusion criterion. For all of these reasons, the results derived from this meta-analyses should be treated with considerable caution.

## Conclusion

To date, evidence from randomized, controlled trials indicated that vitamin D supplementation could increase LDL cholesterol concentrations, but does not appear to significantly affect total cholesterol, HDL cholesterol and triglycerides. The lipid modulating effects of vitamin D supplement should be further investigated through large-scale, randomized trials with adequate doses which can effectively elevated the active form of vitamin D in plasma and with proper population which has hyperlipemia as an inclusion criterion.

## Conflict of interests

The authors declare that they have no competing interests.

## Authors' contributions

HW and NX conducted the literature search, data extraction and quality assessment. YY and DQP conceived the study, its design and drafted the manuscript. All authors read and approved the final manuscript.
